# Delayed Anti-CD3 Therapy Results in Depletion of Alloreactive T Cells and the Dominance of Foxp3^+^CD4^+^ Graft Infiltrating Cells

**DOI:** 10.1111/ajt.12272

**Published:** 2013-06-10

**Authors:** R Goto, S You, M Zaitsu, L Chatenoud, KJ Wood

**Affiliations:** 1 Transplantation Research Immunology Group, Nuffield Department of Surgical Sciences, University of OxfordJohn Radcliffe Hospital, Oxford, United Kingdom; 2 Universite Paris Descartes, Institut National de la Santé et de la Recherche Médicale Unit 1013Paris, France

**Keywords:** graft-infiltrating lymphocytes, heart transplantation, immunosuppressive therapy, regulatory T cells, transplant tolerance

## Abstract

The engineered Fc-nonbinding (crystallizable fragment-nonbinding) CD3 antibody has lower mitogenicity and a precise therapeutic window for disease remission in patients with type 1 diabetes. Before anti-CD3 can be considered for use in transplantation, the most effective timing of treatment relative to transplantation needs to be elucidated. In this study anti-CD3F(ab′)_2_ fragments or saline were administered intravenously for 5 consecutive days (*early*: d1–3 or *delayed*: d3–7) to mice transplanted with a cardiac allograft (H2^b^-to-H2^k^; d0). Survival of allografts was prolonged in mice treated with the *early* protocol (MST = 48 days), but most were rejected by d100. In contrast, in mice treated with the *delayed* protocol allografts continued to survive long term. The *delayed* protocol significantly inhibited donor alloreactivity at d30 as compared to the *early* protocol. A marked increase in Foxp3^+^ T cells (50.3 ± 1.6%) infiltrating the allografts in mice treated with the *delayed* protocol was observed (p < 0.0001 vs. *early* (24.9 ± 2.1%)) at d10; a finding that was maintained in the accepted cardiac allografts at d100. We conclude that the timing of treatment with anti-CD3 therapy is critical for inducing long-term graft survival. Delaying administration effectively inhibits the alloreactivity and promotes the dominance of intragraft Foxp3^+^ T cells allowing long-term graft acceptance.

## Introduction

In transplantation, anti-CD3 therapy was recognized as a powerful therapeutic strategy more than 2 decades ago. OKT3, a mouse monoclonal antibody, efficiently suppressed acute transplant rejection. However its clinical use was hampered by cytokine release syndrome, an undesirable side effect of the antibody’s mitogenicity (1). More recently, Fc-nonbinding CD3 antibodies have been developed. Importantly, Fc-nonbinding or low affinity of Fc binding abrogates or reduces the mitogenic activity (2–4). Data from a phase II clinical trial in type 1 diabetes where Otelixizumab (humanized, mutated Fc, anti-human CD3 antibody) was administered for 6 days after disease onset preserved C-peptide secretion and decreased insulin requirements up to 4 years after antibody therapy (5,6). These data suggest that a short course of Otelixizumab resulted in long-term immunological modulation, supporting data from experimental studies demonstrating that anti-CD3 therapy can result in the re-establishment of tolerance to self-antigens (7,8). In the light of these findings, anti-CD3 therapy may still have use as a therapeutic agent in transplantation with the potential to promote immunological unresponsiveness (4,9–11).

Evidence has been accumulating that anti-CD3 therapy has a precise therapeutic window for promoting a disease remission (11). Previous studies in type 1 diabetes (7) and experimental autoimmune encephalomyelitis (EAE) (12) show that administration of anti-CD3 at disease onset could achieve long-term disease remission, but only after disease onset or the disease priming phase. These data imply that the timing of treatment might be critical for the efficacy of the antibody therapy in organ or cell transplantation. Recently, You and colleagues reported in an islet transplant model, that recipient mice treated with anti-CD3 F(ab′)2 fragments starting on day-1 eventually rejected all grafts, although islet graft survival was significantly prolonged. Whereas, delaying the administration of anti-CD3 therapy 18 out of 21 recipients (85.7%) achieved long-term acceptance of islet allografts (>100 days) (13). In the current study, using a heart transplant model, we have investigated the hypothesis that anti-CD3 therapy will be more effective if initiation of treatment is delayed until after activation of donor reactive T cells has occurred.

In previous studies where T cells from T cell receptor transgenic mice have been tracked *in vivo* as they respond to alloantigen after transplantation, we have found that activation is detectable by 3 days after transplantation (Carvalho-Gaspar and Wood, unpublished data). The data presented herein demonstrate that delaying administration of anti-CD3 therapy until day 3 after transplantation (*delayed* treatment) had a significant impact on long-term graft acceptance. Furthermore, we demonstrate that intragraft, rather than in the draining lymphoid organ, Foxp3^+^ T cells may play an important role for long-term graft acceptance.

## Materials and Methods

### Mice

CBA.Ca (H2^k^), C57BL/10 (B10: H2^b^) and BM3-TCR transgenic male and female mice were provided by and housed in the Biomedical Services Unit at the John Radcliffe Hospital (Oxford, UK). All experiments were performed using protocols approved by the Committee on Animal Care and Ethical Review at the University of Oxford and in accordance with the UK Animals (Scientific Procedures) Act 1986.

### Transplantation

Vascularized heart transplantation (14) was performed as previously described (14). We monitored the heart graft by daily palpation and, when required, direct visualization.

### Treatment protocol

One hundred microliters (50 µg) of anti-CD3 (145-2C11) F(ab′)_2_ fragments were administered intravenously for 5 days perioperatively (from day −1 to 3: *early* 5d protocol) or from 3 days after transplantation for 5 days (from days 3 to 7: *delayed* 5d protocol). Control mice were treated with the same amount of saline. anti-CD3F(ab′)_2_ fragments were produced from the cell line which was kindly provided by J.A. Bluestone (UCSF, San Francisco, CA).

### Flow cytometry

Cell suspensions were prepared from splenocytes following red cell lysis using BD Pharm Lyse (BD Bioscience, San Jose, CA). Graft infiltrating cells were obtained by using collagenase, from *Clostridium histolyticum* (Sigma-Aldrich, Gilligham, UK). Dead cells were excluded by 7AAD staining. Intracellular staining was performed according to the manufacturer’s guidelines (eBioscience, San Diego, CA). For assessing cytokine production, recipient splenocytes were cultured with 100 ng/mL of PMA (Sigma-Aldrich, Inc., St. Louis, MO) and 1 µg/mL of ionomycin (Sigma-Aldrich, Inc., MO) for 6 h. Two microgram per milliliter of BD GolgiStop were added at 2 h before cell harvesting. Cells were stained with Pe-cy7 conjugated CD3 (145-2C11), Pacific Blue conjugated CD4 (*RM4-5*), APC conjugated CD8a (*53-6,7*), PE conjugated CD25 (*PC61.5*), PE conjugated TCR-β (*H57-597*), APC conjugated FoxP3 (*FJK-16s*) and PE conjugated IFNγ (*XMG1.2*) (eBioscience). FACS analysis was carried out with a FACS-Canto II (BD Bioscience) and BD FACSDiva Software Version 6.1.3.

### ELISPOT assay

Recipient splenocytes were co-cultured with irradiated donor splenocytes in MultiScreen 96-Well Plates (Millipore Corporation, Billerica, MA) precoated with an IFNγ antibody (AN18, MABTECH AB, Nacka Strand, Sweden). IFNγ spots were detected by a detection antibody (R4-6A2-biotin, MABTECH AB) and visualized using streptavidin-ALP (MABTECH AB) followed by adding substrate solution (BCIP/NBT, MABTECH AB). Spots were counted using the AID EliSpot Software Version 5.0 (AID GmbH, Ebingerstrasse, Germany).

### Morphometric analysis

Functioning heart allografts retrieved 100 days after transplantation were embedded in OCT-compound (Tissue-Tek, Sakura, The Netherlands) and sectioned at a thickness of 6 µm by using a Bright 5040 Cryostat. These sections were stained with Elastin (VMR International Ltd, Lutterworth, UK) and van Gieson (Raymond A. Lamb Ltd, East Sussex, UK) stain and photographed using light microscopy (Nikon, Tokyo, Japan) and a Coolpix digital camera (Nikon). Intimal expansion rate was calculated as the average from 3 and 5 levels within the heart allograft, with 200 µm of distance between each level. The data from each level were calculated from more than five coronary arteries with more than 75 µm diameter in the heart graft (15). The rate of intimal expansion (current open area/previous open area) was calculated by using Adobe Photoshop CS3 extended (Version 10.0.1) (16).

### Immunohistochemistry (IHC)

Frozen sections (10 µm) of heart graft samples were fixed by cold acetone for 15 min. One percent mouse serum was added for 30 min to block any nonspecific binding sites. After treatment with avidin/biotin block (Vector Laboratories, Inc. Burlingame, CA), biotinylated primary antibodies such as anti-mouse CD3ε (145-2C11, eBioscience), CD4 (L3T4, eBioscience), CD8a (53-6.7, BD Pharmigen, San Diego, CA) and Foxp3 (FJK-16s, eBioscience) were applied for 1 h at room temperature. ABC elite (Vector Laboratories, Inc.) was treated then DAB (Sigma-Aldrich, MO) was added until brown color was visible. Positive cells were confirmed in high magnificent (400×) in light microscopy as previously described (17).

### Statistical analysis

Statistical evaluations were performed with Graphpad Prism software (GraphPad Software, Inc., La Jolla, CA). One-way ANOVA and Student’s t-tests were applied on grouped data. Allograft survival was analyzed using a Kaplan–Meier method and data compared by applying a log-rank test.

## Results

### Delayed administration of αCD3F(ab′)_2_ therapy induces long-term allograft survival

**Figure 1:**
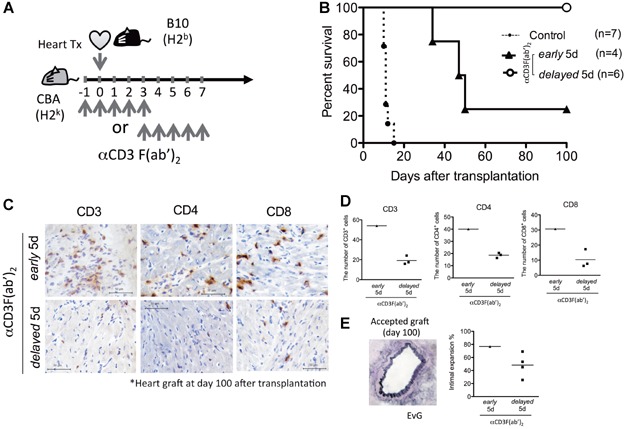
Delayed administration of anti-CD3F(ab′)_2_ therapy after transplantation (days 3–7) results in superior cardiac allograft survival compared to perioperative administration (day −1 to 3). (A and B) anti-CD3F(ab′)_2_ was administered for 5 consecutive days either on day −1 to 3 (*early* protocol) or days 3 to 7 (*delayed* protocol) to CBA recipient mice transplanted with an H2^b^ cardiac allograft on day 0. *p = 0.012, *delayed* versus *early* by log-rank test. (C–E) Heart allografts from the *early* and *delayed* anti-CD3F(ab′)_2_ experimental groups were analyzed at day 100 after transplantation (*early* (n = 1) and *delayed* (n = 3–4)). (C) Representative images from sections stained with biotinylated anti-mouse CD3, CD4 and CD8 antibody respectively are shown (400×). (D) The number of CD3, CD4 and CD8 graft infiltrating cells was quantitated. The scatter plots are the mean number of graft infiltrating cells detected in sections from 4 different levels of the allograft taken at 200 µm intervals, counted in 12 randomly selected areas in high power fields (400×). The vertical bars represent the mean for each treatment group. (E) The images show one of the normal artery sections seen in an accepted allograft from mice treated with the *delayed* anti-CD3 protocol. The scatter plots showed the mean percentage of intimal expansion (IE) rate for mean data of IE rates from five levels as mentioned in Material and Methods. (F and G) anti-CD3F(ab′)_2_ was administered for 5 consecutive days on days 3–7 (*delayed* protocol) to CBA recipient mice transplanted with an aortic allograft from C57Bl10 (H2^b^). The aortic vessel grafts were retrieved from recipient mice at 30 days after transplant and calculated the intimal expansion rate (%) as shown in the Materials and Methods Section. (F) The representative histopathological images were stained with EvG (40×). The intimal expansion areas were shown by red arrows. (G) The degree of intimal expansion was calculated from the data in three separated (200 µm) levels as mentioned in the Materials and Methods section. The data are mean ± SEM values (n = 3–4) from two independent experiments.

Anti-CD3 F(ab′)_2_ fragments (anti-CD3F(ab′)_2_) were used to exclude the impact of Fc binding and assess the effect of anti-CD3 therapy in organ transplantation. Consistent with previously published studies (18–20) anti-CD3F(ab′)_2_ down-regulated cell surface expression of the CD3ε in both activated and resting T cells in a dose-dependent manner (Figure S1). To investigate the hypothesis that delayed administration of anti-CD3 therapy in solid organ transplantation would prevent the allograft rejection more effectively, we compared the impact of treating cardiac allograft recipients with anti-CD3F(ab′)_2_ for 5 consecutive days at the time of transplant (day −1 to 3: *early* protocol) with that of delaying anti-CD3 therapy until day 3 post-transplant (day 3–7: *delayed* protocol) (Figure 1A); the time at which we have shown previously, CD3^+^ T cells begin to infiltrate the cardiac allograft (21) and when Foxp3^−^CD25^+^CD4^+^ T cells are detectable within the heart allograft (day 4 after transplantation; Figure S2).

CBA.Ca (H2^k^) recipient control mice, treated with saline intravenously, rejected C57/B10 (B10: H2^b^) heart allografts acutely (MST = 11 days). When anti-CD3 was administered in accordance with the *early* protocol (day −1 to 3; Figure 1A) allograft survival was prolonged, although three of four allografts were rejected (MST = 48 days; Figure 1B). In contrast, when initiation of anti-CD3 therapy was delayed until 3 days after transplantation and continued for 5 days, *delayed* protocol (day 3–8; Figure 1A), all heart allografts were functioning at 100 days (MST > 100 days; Figure 1B). These data suggest that the critical period for the therapeutic efficacy of anti-CD3 therapy is after transplantation (Figure 1B).

Allografts functioning at 100 days in each experimental group were analyzed by histopathology (n = 1 *early* protocol and n = 3–6 *delayed* protocol). The myocardial architecture was found to be well-preserved in the *delayed* treatment group with lower numbers of graft infiltrating CD3, CD4 and CD8 T cells present (Figure 1C and D). In addition, there was a trend towards changes in vessel architecture being less pronounced in mice treated with the *delayed* versus *early* protocol, although transplant arteriosclerosis was not absent in any of the heart allografts (Figure 1E).

### Alloantigen reactivity and the number of CD3^+^ T cells was similar in mice treated with the early and delayed anti-CD3F(ab′)_2_ treatment protocols 10 days after transplantation

**Figure 2:**
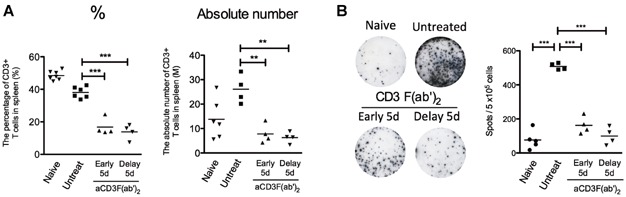
Both *early* and *delayed* anti-CD3F(ab′)_2_ treatment prevented alloimmune responses during the early posttransplant period. (A and B) The spleens were retrieved from recipient mice treated with either saline (n = 3-6); the *early* (n = 4) or *delayed* (n = 4) anti-CD3F(ab′)_2_ protocols at day 10 after transplantation. Data were pooled from more than two independent experiments. (A) The percentage and absolute number (×10^6^ (Million, M)) of CD3^+^ T cells in spleen are shown. CD3 expression after gating lymph gate in FCS/SSC was assessed by FACS. Dead cells were excluded by 7AAD staining. ***p < 0.0001, **p < 0.005. (B) 5 × 10^5^ recipient splenocytes were co-cultured with 7.5 × 10^5^ of irradiated donor C57BL/10 splenocytes for 20 h in anti-IFNγ antibody precoated ELISPOT plate as mentioned in the Materials and Methods section. The spots were counted by ELISPOT reader. The representative images were shown from two independent experiments (left). The each scatter plots are mean for duplicate data. The vertical bars are mean for mice treated with each protocol. ***p < 0.0001, n = 4 per each group.

Next, to examine the mechanisms underpinning the beneficial effect of the *delayed* anti-CD3 protocol, we investigated the impact of therapy on immune responses *in vivo* 10 days after transplantation. As expected, treatment with anti-CD3F(ab′)_2_ significantly reduced the percentage and absolute number of CD3^+^ T cells detectable in the spleen (Figure 2A). However, no significant differences in the number of CD3^+^ cells were detected in mice treated with the early and *delayed* protocols (Figure 2A). Further, to exclude the possibility that CD3ε on the surface of T cells was masked by anti-CD3 F(ab)′_2_, the percentages and absolute numbers of CD4^+^ and CD8^+^ lymphocytes were investigated. The significant reduction of percentages and numbers of CD4^+^ and CD8+ T cells was found in both the *early* and *delayed* protocols. Consistent with these data (Figure 2A), the ability of T cells to respond to donor alloantigens, investigated by co-culturing splenocytes isolated from mice 10 days after transplantation with irradiated donor leukocytes and assessed by IFNγ ELISPOT, was inhibited by εCD3F(ab′)2 therapy using either the *early* or *delayed* protocol (Figure 2B). The frequency of the IFNγ producing cells 10 days after transplantation tended to be lower in mice treated with the *delayed* protocol compared to the *early* protocol, but the differences were not statistically significant (Figure 2B). These data indicated that a 5-day course of treatment with anti-CD3F(ab′)_2_ either perioperatively or delayed until d3 after transplantation effectively prevented immune reactivity to donor alloantigens within the lymphoid organ draining the allograft.

### The delayed protocol effectively induces an alloimmune suppression. Meanwhile, alloreactive T cells emerge after repopulation of CD3^+^ T cells in the early protocol

**Figure 3:**
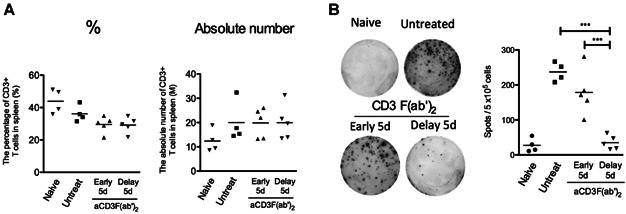
The alloreactivity after repopulation of CD3 T cells was sufficiently suppressed by the *delayed* anti-CD3F(ab′)_2_ *treatment* but does not in the *early* protocol. (A and B) The spleens were retrieved from recipient mice at day 30 after transplantation and analyzed as indicated in Figure 2. Data were pooled from more than two independent experiments. (A) The percentage and absolute number of CD3^+^ T cells in spleen are shown. (B) IFNγ ELISPOT assay was performed as described in Figure 2 legend and the Materials and Methods section. The representative images were shown from two independent experiments (left). The scatter plots are mean for duplicate data. The vertical bars are mean for mice treated with each protocol. ***p < 0.0001, n = 4 per each group.

In contrast to the data observed 10 days after transplantation (Figure 2AB), by day 30 when the number of CD3^+^ T cells was back to base line levels (Figure 3A), IFNγ production by the alloreactive T cells was detectable in the *early* treatment group (Figure 3B) but not in the *delayed* protocol group completely when cells were stimulated with donor irradiated lymphocytes *ex vivo* (p < 0.0001 vs. *early* and control group, Figure 3B). These data suggest that the *early* protocol prevents the rejection in the perioperative period by reducing the total number of CD3^+^ T cells but does not inhibit the response to donor alloantigen specifically. In contrast, the *delayed* treatment protocol effectively inhibits the donor alloantigen specific response.

### A significant increase in intragraft Foxp3^+^CD4^+^ T cells was observed in mice treated with the delayed αCD3F(ab′)_2_ protocol

**Figure 4:**
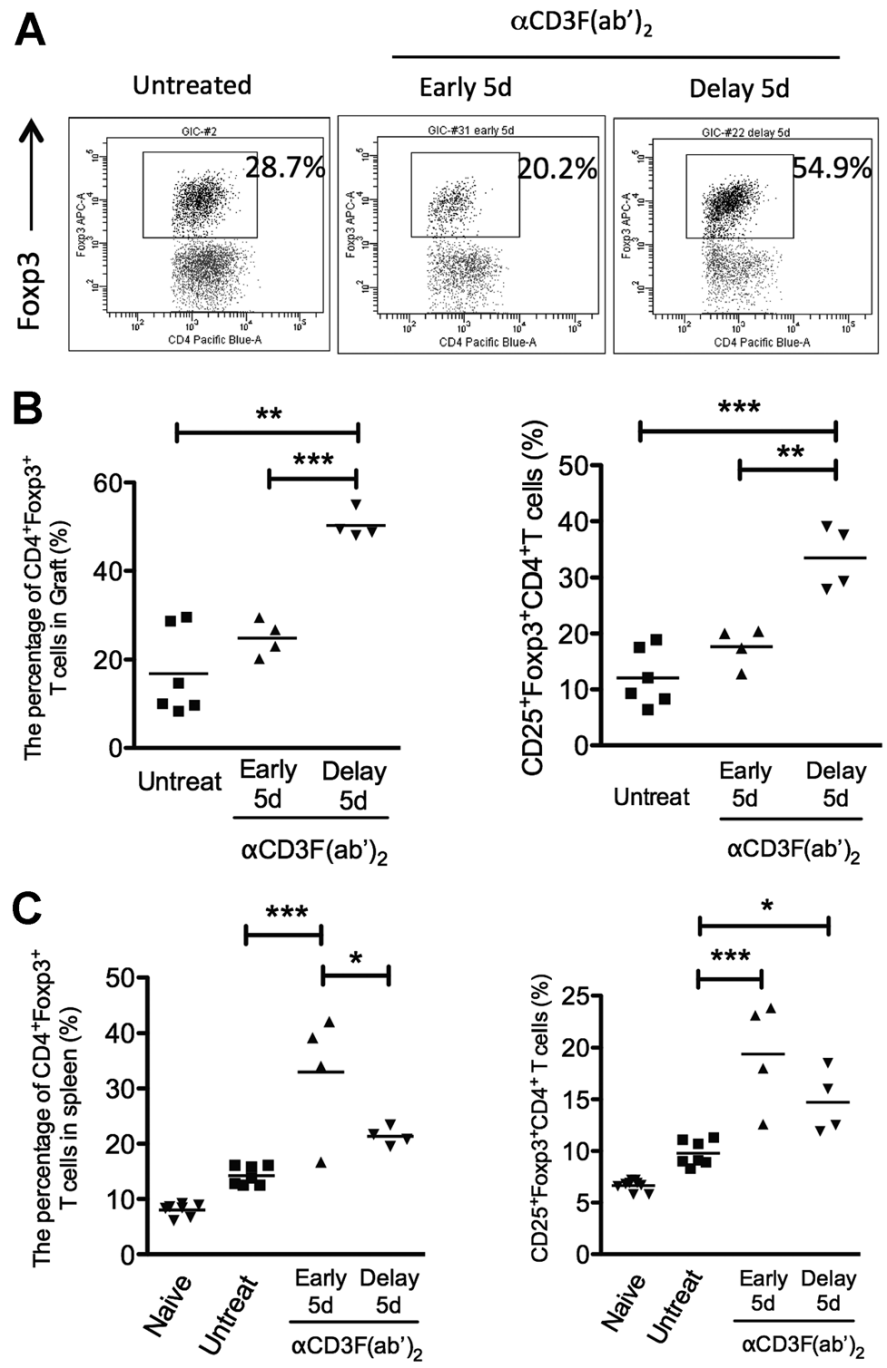
Delayed anti-CD3 therapy increases the Foxp3^+^CD4^+^ T cells infiltrating allograft. (A–C) Graft infiltrating cells and splenocytes were obtained 10 days after transplantation from the recipient mice that were either untreated or treated with *early* or *delayed* anti-CD3F(ab′)_2_ treatment for 5 days. (A) The representative images show the Foxp3 positive graft infiltrating cells after gating on CD4^+^ T cells. (B and C) The dot plots demonstrate the percentage of Foxp3^+^ (left) and CD25^+^Foxp3^+^ (right) T cells after gating CD4 in graft (B) and spleen (C) respectively. The data were ***pooled from two independent experiments. *p < 0.05, **p < 0.001, ***p < 0.0001.

Further, to investigate the hypothesis that the composition of the graft infiltrating cells was altered in mice treated with the *delayed* protocol, we determined the number of Foxp3^+^CD4^+^ T cells infiltrating the heart allograft in the early posttransplant period. Importantly, approximately half of CD4^+^ T cells (mean = 50.3%) within the allografts of mice treated with the *delayed* protocol expressed Foxp3, compared to a mean of 24.9% and 16.8% of Foxp3^+^CD4^+^ T cells in the grafts retrieved from mice treated with the *early* protocol or controls (***p < 0.0001 vs. *early* protocol, Figure 4A and B, left). Furthermore, the expression of CD25 by these intragraft Foxp3^+^ T cells was significantly increased in the *delayed* treatment group compared to those in the *early* or untreated control groups (Figure 4B, right). Interestingly, in the spleen, the number of Foxp3^+^ cells and CD25 expression by Foxp3^+^CD4^+^ T cells were the inverse of that found infiltrating the allografts (Figure 4C, right and left). The spleens from mice treated with the *early* 5d protocol had a significantly increased percentage of Foxp3^+^ cells as compared to that in untreated controls and even the *delayed* treatment group (Figure 4C, left).

### Intragraft Foxp3^+^CD4^+^ T cells were maintained at a higher level in mice treated with the delayed CD3F(ab′)_2_ protocol

**Figure 5:**
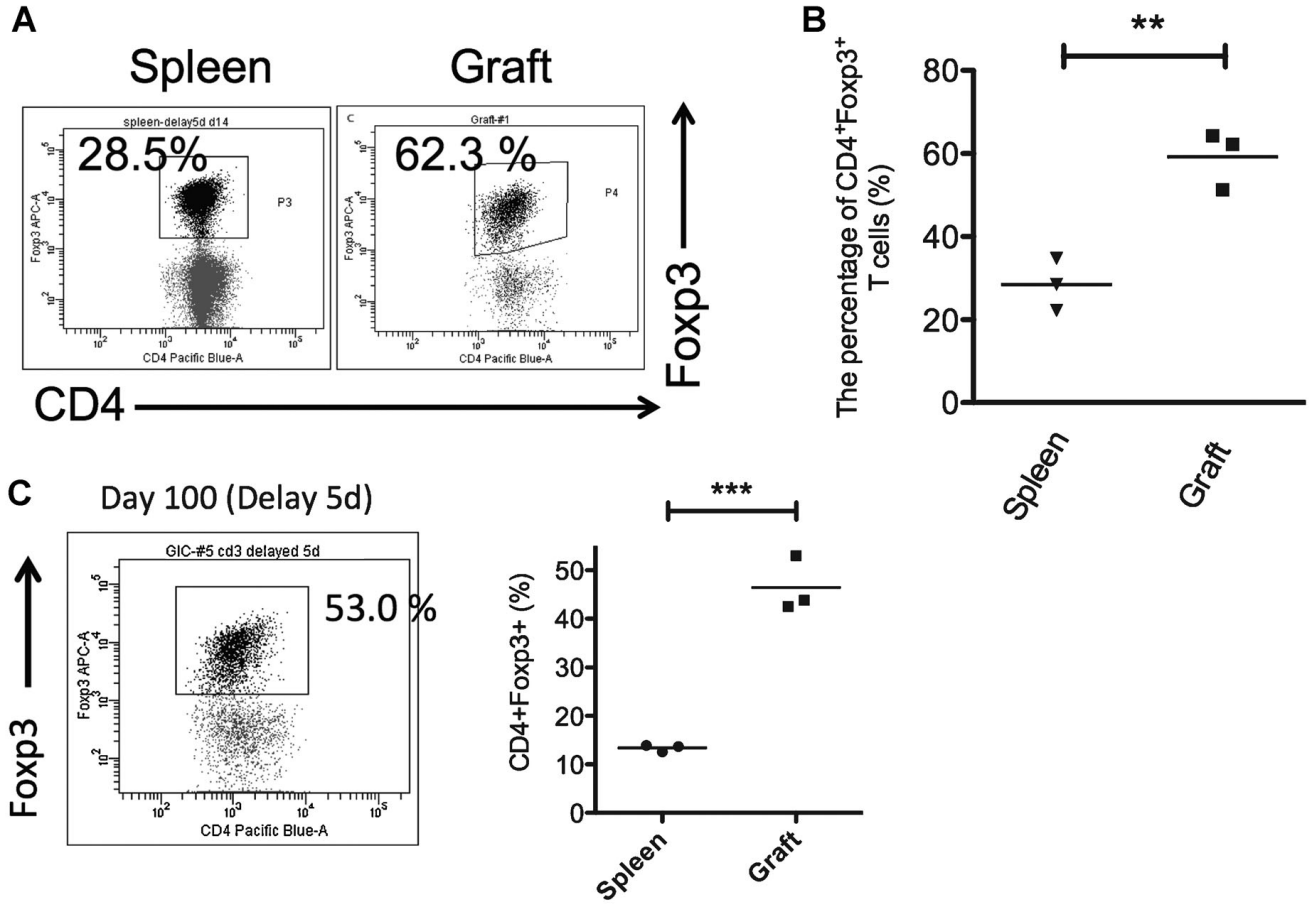
The splenic and intragraft Foxp3^+^CD4^+^ cells at 14 and 100 days posttransplantation. (A–C) Graft infiltrating cells and splenocytes were obtained from the recipient mice treated with the *delayed* anti-CD3F(ab′)_2_ protocol on day 14 (A and B) and day 100 (C) after transplantation. The representative image shows Foxp3 positive cells after gating CD4 (A and C). The dot plots demonstrate the percentage of CD4^+^Foxp3^+^ T cells in spleen and graft, respectively (B and C). **p < 0.005, ***p < 0.001.

An increase in Foxp3^+^CD4^+^ T cells in the peripheral lymph node was found to be maximal 6 days posttreatment (22). In this study, day 10 after transplantation is 7 days after completion of treatment with anti-CD3F(ab′)_2_ for *early* protocol but only 3 days for delayed protocol. Therefore, to assess mice treated with either protocol at the same time point after antibody treatment, we investigated splenic Foxp3^+^CD4^+^ T cells 14 days after transplantation, that is, at 7 days after completion of *delayed* protocol treatment. A higher percentage of Foxp3^+^CD4^+^ T cells was found in the spleen (Figure 5A and B) compared to that observed at day 10 after transplantation iN mice treated with the *delayed* protocol (Figure 4C). This proportion was similar to that observed in the spleens of mice treated with the *early* protocol at 7 days after completion of treatment (Figure 4C), suggesting that an increase in splenic Foxp3^+^CD4^+^ T cells was associated with completion of treatment with αCD3F(ab′)_2_. More importantly intragraft Foxp3^+^CD4^+^ T cells were maintained or present at a higher level at day 14 after transplantation in mice treated with the *delayed* protocol (Figure 5A and B). Furthermore at 100 days after transplantation, Foxp3^+^CD4^+^ remained predominant within the allograft, but not spleen in mice treated with the *delayed* protocol (Figure 5C). These data suggest that delaying the administration of anti-CD3F(ab′)_2_ treatment significantly increased the number of intragraft Foxp3^+^ T cells; a finding that may be important for inducing long-term allograft acceptance.

## Discussion

The data from this study demonstrate that Fc-nonbinding anti-CD3 therapy is applicable to organ transplantation; a *delayed* short course of treatment with anti-CD3 F(ab′)_2_ (5 days starting on d3 posttransplant) resulted in long-term survival of fully allogeneic heart allografts in mice (Figure 1B). Consistent with the findings reported here, You and colleagues have clearly shown that the *delayed* initiation of anti-CD3 antibody treatment (from day 7) effectively promotes acceptance of allogeneic islet grafts (13). Interestingly, the timing of initiation of the anti-CD3 treatment protocol was different between heart and islet allografts, that is, 3 and 7 days posttransplantation respectively. It seems that the efficacy of different timing of administration of anti-CD3 therapy is associated with the difference in the kinetics of graft infiltration between primarily vascularized and secondarily vascularized transplants. Our data using a heart allograft model showed that graft infiltration by T cells is initiated at day 3 after transplantation (Figure S2) and by day 4 activated CD4^+^ T cells (CD4^+^CD25^+^Foxp3^−^ T cells) are present within the graft (Figure S2). Fully allogeneic heart allografts are completely rejected by day 11 in this strain combination. In contrast, You et al. found that the MST for allogeneic islet grafts was 18.8 ± 0.9 days, suggesting that the graft infiltration is initiated at a later time point. These data support the hypothesis that the ability of anti-CD3 treatment to induce specific unresponsiveness to alloantigens requires T cell priming, the kinetics of which varies with different types of transplant.

In addition to differences from graft vascularization, the heart allograft transplant model has an advantageous point for the observation of vascular changes in the allograft. A short course of low-dose anti-CD3 monotherapy could not completely prevent the development of transplant arteriosclerosis in vascularized heart (Figure 1E) or aorta allografts (data not shown) using the protocol developed for this study. Further work is required to determine if anti-CD3 in combination with other agents could impact transplant arteriosclerosis. It suggests that anti-CD3 therapy merits further consideration for use in clinical transplantation in combination with other immunosuppressive agents. In terms of combination therapy, cycloporin A was found to abolish the tolerogenic effect of anti-CD3 antibodies in NOD mice, probably because this calcineurin inhibitor inhibits T cell activation and apoptosis, both key pathways in the mechanisms of action of delayed anti-CD3 therapy (7). It has also been shown that rapamycin abrogates and reverses anti-CD3 antibody-induced diabetes remission (23). However, in relation to this latter point, caution has to be taken in interpreting these data as it is known that (i) rapamycin has a potent toxic effect on pancreatic beta cells and (ii) rapamycin withdrawal leads to a return to normoglycemia, that is, remission. Thus, the negative immunological effect of rapamycin on delayed administration of anti-CD3 antibody therapy remains to be determined. Considering the favorable effect of regulatory T cells on allograft rejection (24), cellular therapy using a regulatory immune cell population may be an attractive candidate for consideration, in combination with anti-CD3 therapy (25).

Taking into account that this was mono-therapy, and a short course of treatment, these data are comparable with those of other biological immunosuppressants such as CTLA-4Ig and anti-CD154 (26,27) More interestingly, our data demonstrate that the *delayed* treatment with anti-CD3 is critical for inducing long-term allograft acceptance as it enables targeting of activated T cells as they respond to donor alloantigens. These data suggest that the perioperative treatment with anti-CD3 (day −1 to day 3) while immunosuppressive, does not have a positive impact on long-term graft outcome. Conventionally, with only a few exceptions, administration of immunosuppressive agents immediately after or before grafting has been considered to be optimal (28,29). On the other hand, excessive immunosuppression and/or blockade of alloantigen recognition (signal 1) at the time of transplantation has been shown to prevent the induction of operational tolerance in organ transplant recipients (30). Indeed, inhibition of T cell activation by calcineurin inhibitors abrogated mechanisms such as activation induced cell death (AICD) (11,31,32). Our data are consistent with the findings that anti-CD3 therapy induces the loss of CD28–CD80 clusters in the T cell–DC interface (33,34). Further CTLA-4Ig treatment has been reported to have a similar effect in organ transplantation (35). Moreover, given that many immunosuppressants target T cell activation, it is possible that our data with anti-CD3, and earlier studies with CTLA-4Ig, indicate an opportunity to reconsider the timing of some treatment protocols in the transplant setting.

We proposed the hypothesis that *delayed* treatment with anti-CD3 may induce the depletion of alloreactive T cells more profoundly than perioperative treatment through AICD, thereby preventing T cell mediated rejection (34). Previous work has shown that antibodies induce apoptosis of activated T cells through alterations in TCR-mediated signal transduction (31), a finding confirmed by using OVA-specific OT1 transgenic T cells where a significantly higher percentage of annexin V+ OT-1 cells was found in draining LN and spleen in anti-CD3 treated mice (13). Importantly, You and colleagues demonstrated that proliferated alloreactive T cells precipitate apoptosis more profoundly, a finding consistent with our data *in vitro* where anti-CD3 therapy was found to promote apoptosis of TCR+ lymphocytes after alloantigen stimulation (data not shown).

However, examining donor alloreactivity on day 10 after heart transplantation, we found no significant difference between the early and delayed protocols (Figure 2B). At this time point, while both treatment protocols significantly reduced the number of CD3^+^ T cells, the early protocol did not suppress the immune response to alloantigen, as demonstrated by IFNγ production after restimulation *in vitro*, but the delayed protocol inhibited donor alloreactivity completely 30 days after transplantation; a time point when anti-CD3 antibody was no longer detectable in the recipient and the number of CD3^+^ T cells had recovered (Figure 3). These data suggest that, at least in part, the administration of anti-CD3 therapy after alloantigen priming induces apoptosis of alloreactive T cells promoting heart allograft survival. In terms of donor alloantigen specificity of the unresponsiveness state achieved by delayed anti-CD3 therapy, T cells present in the periphery of mice 30 days after transplantation retained the capacity to respond, as demonstrated by their ability to secrete IFNγ upon stimulation with PMA/ionomycin. However, they did not respond to donor alloantigen stimulation. Taken together with *in vivo* data from a study by You and colleagues using an islet transplant model, where second islet grafts from original donors were accepted but islets from third-party donors (C3H) were rejected by mice treated with delayed anti-CD3 therapy (36), we conclude that delayed anti-CD3 therapy can lead to specific immunological unresponsiveness to a cardiac allograft.

At this time point, while both treatment protocols significantly reduced the number of CD3^+^ T cells, the early protocol did not suppress the immune response to alloantigen, as demonstrated by IFNγ production after restimulation *in vitro*, but the delayed protocol inhibited donor alloreactivity completely 30 days after transplantation; a time point when anti-CD3 antibody was no longer detectable in the recipient and the number of CD3^+^ T cells had recovered (Figure 3). These data suggest that, at least in part, the administration of anti-CD3 therapy after alloantigen priming induces apoptosis of alloreactive T cells promoting heart allograft survival.

We also investigated the distinct differences observed in the percentage of intragraft Foxp3^+^CD4^+^ cells between the treatment strategies (Figure 4AB). It seems highly likely that Foxp3^+^CD4^+^ T cells that are associated with immunoregulation contribute to the differences we observed in graft survival (Figure 1). Notably, intragraft Foxp3^+^CD4^+^ cells were maintained at a higher level—for example, approximately 50% of CD4^+^ T cells at both day 14 and 100 after transplantation in mice treated with the *delayed* anti-CD3 protocol (Figure 5). Thus *delayed* treatment with anti-CD3 results in the dominance of intragraft Foxp3^+^CD4^+^ T cells that have the potential to promote allograft acceptance.

Resident intragraft regulatory T cells have been reported to chaperone cells that retain the capacity to reject the allograft. For example, when intra-graft Foxp3^+^ regulatory T cells were depleted from functioning allografts they were immediately rejected without the need for infusion of additional effector T cells (37). Indeed, in this study the Foxp3^+^ cells detected in the heart allografts 100 days after transplantation following treatment with the delayed anti-CD3 protocol were located within the same microenvironment as other Foxp3^−^ graft infiltrating lymphocytes. This suggests that Foxp3^+^ T cells may be acting as chaperones in this setting and controlling the activity of other lymphocytes. Foxp3^+^CD4^+^CD25^+^T cells were also present in the grafts of recipients treated with the *delayed* anti-CD3F(ab′)_2_ protocol at earlier time points after transplantation (Figure 4B, right). Regulatory T cells present in the graft have the potential to be repeatedly or chronically stimulated by donor alloantigen, a process that has been shown to make regulation more potent (38). Furthermore, activated regulatory T cells that are constitutively cycling, express higher levels of Foxp3 and CTLA-4, and are more potent suppressors (38,39). Taken together, these data suggest that regulatory T cells present in surviving allografts might be more efficient at protecting the allograft from damage. Although *delayed* anti-CD3 therapy results in the presence of intragraft Foxp3^+^ T cells, the precise mechanism underpinning the effectiveness of delaying treatment until 3 days after transplantation remains unclear. To date, there is no evidence that anti-CD3 can convert conventional T cells into Treg in the periphery, rather that the increase in Foxp3^+^Treg is a consequence of pre-existing Tregs being resistant to anti-CD3-induced cell death (22,40). Thus, we propose that Tregs, pre-existing in the graft at the time of the delayed treatment, resist CD3 antibody-induced apoptosis and therefore exhibit *in situ* a potent regulatory control on effector T cells, limiting their expansion and alloreactivity.

In this study, we showed that anti-CD3 therapy can not only prevent allograft rejection but also lead to long-term allograft survival when the timing of anti-CD3 administration is delayed until 3 days after transplantation. Due to the availability of humanized CD3 antibodies, further consideration of using anti-CD3 therapy in transplantation is warranted.

## Abbreviations

Fc: crystallizable fragment

## Notes

Figure S1 **αCD3 F(ab′)**_**2**_ **treatment down-regulated cell surface expression of CD3ε on activated and resting T cells** ***in vitro***. Lymphocytes retrieved from the spleens and lymph nodes of BM3 TCR transgenic mice were co-cultured with anti-CD3 F(ab′)_2_ fragments for 2 days at the concentrations indicated in the figure. Cells were stimulated in the presence or absence of Lectin from *Phaseolus vulgaris* (Sigma-Aldrich, Inc., St. Louis, MO) so-called PHA (5 μg/mL). After 2 days, harvested cells were stained with PE conjugated TCR-β, PEcy7 conjugated CD3ε and 7AAD and assessed by FACS-Canto. Data are representative of two independent experiments.

Figure S2 **Graft infiltrating cells during early transplant period**. Lymphocytes were obtained from the allograft (H2^d^) at 3 and 4 days after cardiac transplantation. The cells were stained with PE conjugated with FITC conjugated TCR-β, PEcy7 conjugated CD3ε, PB conjugated CD4, PE conjugated CD25, APC conjugated Foxp3 and 7AAD by intracellular staining techniques and assessed by FACO-Canto. Data are representative of two independent experiments. **p < 0.001.
