# Supplementary material for: Delayed Anti-CD3 Therapy Results in Depletion of Alloreactive T Cells and the Dominance of Foxp3+CD4+ Graft Infiltrating Cells
Source: Am J Transplant. 2013 Jun 10;13(7):1655–64. doi: 10.1111/ajt.12272 (PMC3790953; doi:10.1111/ajt.12272)
Supplement: Figure S1 — αCD3 F(ab′)2 treatment down-regulated cell surface expression of CD3ε on activated and resting T cells in vitro. Lymphocytes retrieved from the spleens and lymph nodes of BM3 TCR transgenic mice were co-cultured with anti-CD3 F(ab′)2 fragments for 2 days at the concentrations indicated in the figure. Cells were stimulated in the presence or absence of Lectin from Phaseolus vulgaris (Sigma-Aldrich, Inc., St. Louis, MO) so-called PHA (5 μg/mL). After 2 days, harvested cells were stained with PE conjugated TCR-β, PEcy7 conjugated CD3ε and 7AAD and assessed by FACS-Canto. Data are representative of two independent experiments. [file ajt0013-1655-SD1.pdf]

Figure S1

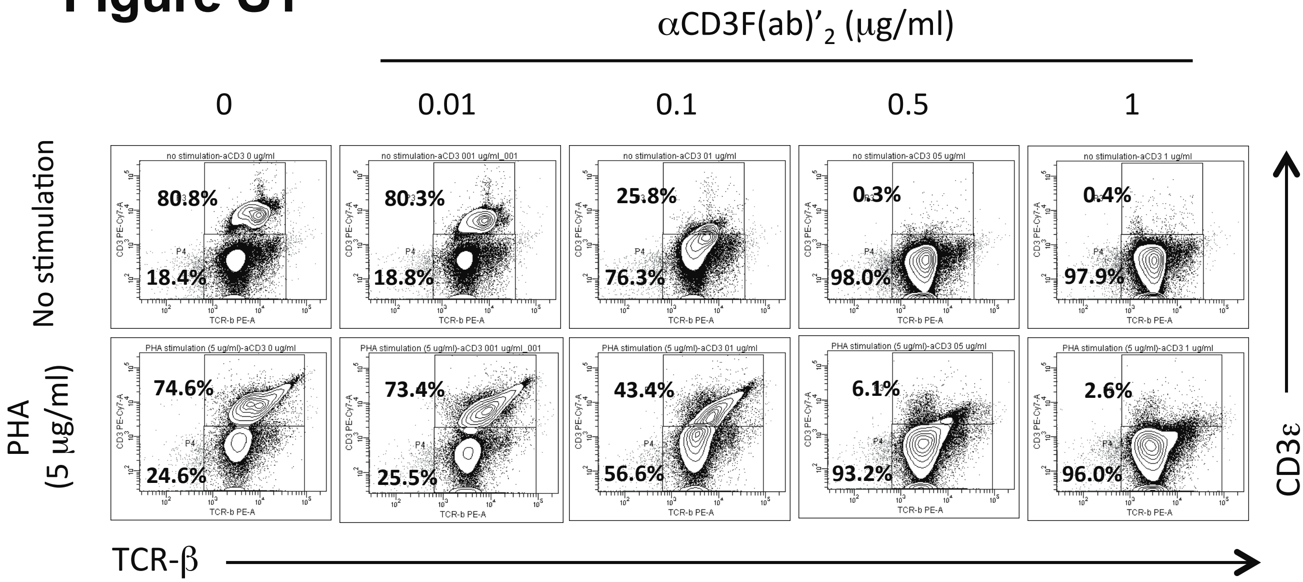

Figure S1: αCD3 F(ab')$_2$ treatment down-regulated cell surface expression of CD3ε on activated and resting T cells *in vitro*. Lymphocytes retrieved from the spleens and lymph nodes of BM3 TCR transgenic mice, were cocultured with αCD3F(ab')$_2$ fragments for 2 days at the concentrations indicated in the figure. Cells were stimulated in the presence or absence of Lectin from Pharseolus vulgaris (Sigma-Aldrich, Inc. Saint Louis, MO) so-called PHA (5 μg/ml). After 2 days, harvested cells were stained with PE conjugated TCR-β, PEcy7 conjugated CD3ε and 7AAD and assessed by FACS-Canto. Data are representative of 2 independent experiments.
