# Supplementary material for: Delayed Anti-CD3 Therapy Results in Depletion of Alloreactive T Cells and the Dominance of Foxp3+CD4+ Graft Infiltrating Cells
Source: Am J Transplant. 2013 Jun 10;13(7):1655–64. doi: 10.1111/ajt.12272 (PMC3790953; doi:10.1111/ajt.12272)
Supplement: Figure S2 — Graft infiltrating cells during early transplant period. Lymphocytes were obtained from the allograft (H2d) at 3 and 4 days after cardiac transplantation. The cells were stained with PE conjugated with FITC conjugated TCR-β, PEcy7 conjugated CD3ε, PB conjugated CD4, PE conjugated CD25, APC conjugated Foxp3 and 7AAD by intracellular staining techniques and assessed by FACO-Canto. Data are representative of two independent experiments. **p < 0.001. [file ajt0013-1655-SD2.pdf]

Figure S2

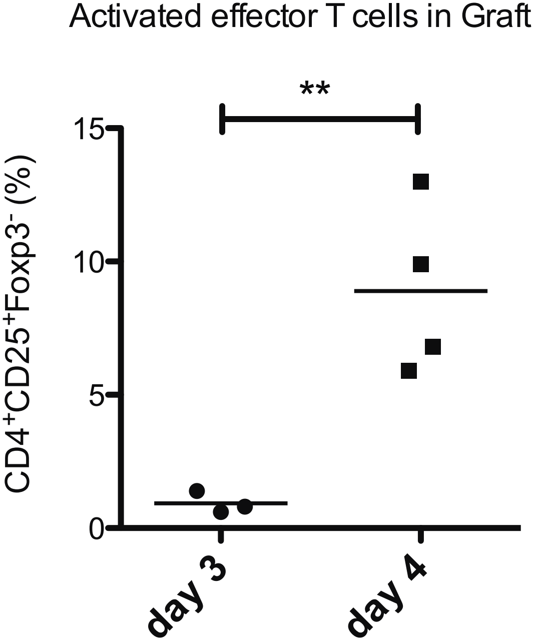

Figure S2: Graft infiltrating cells during early transplant period. Lymphocytes were obtained from the allograft ($H2^d$) at 3 and 4 days after cardiac transplantation. The cells were stained with PE conjugated with FITC conjugated TCR-β, PEcy7 conjugated CD3ε, PB conjugated CD4, PE conjugated CD25, APC conjugated Foxp3 and 7AAD by intracellular staining techniques and assessed by FACS-Canto. Data are representative of 2 independent experiments. **p<0.001.
